# Supplementary material for: Chromothripsis during telomere crisis is independent of NHEJ, and consistent with a replicative origin
Source: Genome Res. 2019 May;29(5):737–49. doi: 10.1101/gr.240705.118 (PMC6499312; doi:10.1101/gr.240705.118)
Supplement: Supplemental Material [file supp_gr.240705.118_Supplemental_file_1.zip › contigs/annotated_contigs/DB53/contig.4.DB53_length_490_mean_cov_5.62448979592.docx]

**DB53_length_490_mean_cov_5.62448979592**

```
CCACCCATCCACTTACCCATCTATCCCTCCATCCACCCATCCAC|TCCTCCATCCACCG|ATCCACCTCTCCATCCACCCCTCCATCCA
 >chr9:136703596-136703640 - E=8e-03 p=0e+00               >chr15:40285342-40285381 + E
CCCAC|CCATC|TACCCATCCCTCCCTCCATCCCTCTATCCAACCATCCATCCACCCATCTATCCCTCCATTCACATATCCATCCTTCC
=8e-03 p=0e+00 >chr7:155844667-155844855 + E=1e-98
ATCCACCCATCTATCCCTCCATCCACCCATCCACTTACCCATCTATCCCTCCATCCACCCATCCATTCCTCCATCTACCGATTCATCTC

TCCATCCACCCCTTCAT|TAAGCCTTT|CCTGAGCACGTTTTATGTTTGTTCACCCAGAAACAGCTCCTGTTACTAGCTCATCCCCTCA
                           >chr8:135655962-135656151 + E=4e-87
GAATGGGGGCGCCTTTTGTAATCTGCACAAAGGTCCCTATGTGCCAGCCAAGGCTCCAGCAGTCACATGAAAGCTCCAGGCATGGAGCC

TAAGTCTGAGTCCAAGACGTGTGCCGCTTCCACTACACT|CCGGGGCTGGCC
```
